# Potential Distribution and Niche Differentiation of *Spodoptera frugiperda* in Africa

**DOI:** 10.3390/insects11060383

**Published:** 2020-06-21

**Authors:** Jingyu Fan, Pengxiang Wu, Tianqi Tian, Qilin Ren, Muhammad Haseeb, Runzhi Zhang

**Affiliations:** 1 Institute of Zoology, Chinese Academy of Sciences, No 1-5, Beichen West Rd. Chaoyang, Beijing 100101, China; fanjingyu@ioz.ac.cn (J.F.); wupengxiang@ioz.ac.cn (P.W.); tiantianqi15@mails.ucas.edu.cn (T.T.); qlren94@163.com (Q.R.); 2 College of Life Science, University of Chinese Academy of Sciences, No.19(A) Yuquan Road, Shijingshan District, Beijing 100049, China; 3 Institute of Ecology and Geography, Chinese Academy of Sciences, 818 South Beijing Road, Urumqi, Xinjiang 830011, China; 4 Institute of Entomology, Guizhou University, Huaqi, Guiyang 550025, China; 5 Center for Biological Control, College of Agriculture and Food Sciences, Florida Agricultural and Mechanical University, Tallahassee, FL 32307, USA; muhammad.haseeb@famu.edu

**Keywords:** fall armyworm, maize pest, ecological niche model, invasion, risk analysis

## Abstract

The fall armyworm, *Spodoptera frugiperda* (J.E. Smith) is a serious agricultural pest. The species originates from the tropical and subtropical regions of the Americas and has now become established in many countries. Its strong migratory ability is the key factor in the rapidly expanding range of *S. frugiperda* in Africa, where food security faces unprecedented challenges. Exploring potential distributions and niche differentiation of *S. frugiperda* could provide new insights into the nature of climate niche shifts and our ability to anticipate further invasions. In this study, the occurrence population records (native, source, global, and African) and environmental variables of *S. frugiperda* were selected to fit ecological niche models (ENMs), with an evaluation of niche conservatism during its invasion of Africa. The results showed that the potential distributions of *S. frugiperda* are mainly in tropical and subtropical areas in Africa. The climate spaces occupied by its native population and introduced African population broadly overlap. Although, climate niches were conserved during invasion of Africa, many climate spaces were unoccupied, suggesting a high remaining invasion potential in Africa. The selection of the biogeographic realm is an important factor in model construction, and has a great influence on the transferability of the models. Indeed, the global model produced the best performance, following the source and native models.

## 1. Introduction

Globalization and changes in trade have increased accidental introductions of invasive species around the world. When established in the novel environment(s), they have the ability to endanger native species communities and composition [1], and have a detrimental effect on agricultural production systems [2,3]. In recent years, the shipping system has become an important driving force for the spread of invasive pests. The emerging global shipping network could lead to a 3- to 20-fold increase in global invasion risk [4]. In general, outbreaks of invasive pests tend to be very difficult to control [5]. The most effective way is to prevent their initial invasion or to restrain future expansion rather than to limit their outbreaks. Exploring ecological dimensions and estimating the potential geographic distribution of invasive species can provide a reference for risk analysis and integrated pest management.

The fall armyworm, *Spodoptera frugiperda* (J. E. Smith) (Lepidoptera: Noctuidae) is native to the tropical and subtropical regions of the Americas [6], where it is recognized as one of the most damaging crop pests with fast dispersal rates [7]. The pest prefers to live in relatively warm and moist areas, as it cannot overwinter and survive in cold temperatures [8]. Every year, adult moths in tropical areas move northwards to temperate regions, threatening areas outside their native ranges [9]. An earlier study indicated that *S. frugiperda* occurrences in south Florida (27 °C) and south Texas (31 °C) were a year-round phenomenon [10]. In July, 2018, the pest was confirmed to be present in India and Yemen. By December 2018, it was reported in Bangladesh, Sri Lanka and Thailand. As of June 2019, it has been reported in Myanmar, China, Indonesia, Laos, Malaysia, Viet Nam, Egypt and the Republic of Korea. Japan reported the presence of *S. frugiperda* in July 2019. It was officially reported in Australia and Mauritania in February 2020, and in Timor-Leste in March 2020 [11].

Every year, *S. frugiperda* causes up to a 34% reduction in maize yield [12] and economic losses of up to $400 million in Brazil alone [13]. Maize is one of the most important staple food crops in Africa for commerce and farm households [14]. *S. frugiperda* was first reported in maize fields in Nigeria [15]. Subsequently, it spread to west African countries including São Tomé and Príncipe in April, and later in Bénin, Ghana and Togo in June. In 2018, it was found in 44 African countries, covering more than 25 million km^2^ [16], where it posed a serious threat to agricultural production. Studies have shown that *S. frugiperda* entered Africa as a stowaway on a passenger flight through trade [17]. In Africa, i.e., the species’ first major introduction into the Eastern Hemisphere, it showed an obvious trend of population growth and distribution expansion. The pest has rapidly spread throughout most of the maize-producing areas of Africa since it was introduced and established in 2016, causing potential economic losses of between US $2.481 billion and $6.187 billion per year [18]. Indeed, the pest has now invaded nearly 100 countries around the world, and continues to find novel ecological regions where it is threating agricultural production systems, potentially leading to food shortages [19].

Ecological niche modeling (ENM) seeks to characterize the ecological requirements of a given species using environmental variables associated with occurrence data, from which it is possible identify where suitable environmental space are distributed [20]. The global model is built on the basis of a population nonequilibrium state, and tends to identify potential areas that cluster around observed points [21]. The native model is built on the basis of population equilibrium to calibrate the niche model in native areas. If fundamental niches differ between the native population and invasion populations, models based on the entire native range of a species may miscalculate a species’ potential distribution. Therefore, an earlier study suggested that a source population and introduction locations could be pooled together to establish a model, which could be a potential method for overcoming this limitation [22].

Realized niche shifts are commonly investigated by examining changes in occupied regions of environmental space or by testing how well ENMs parameterized on a species’ native range can predict its invaded range [23,24]. It can effectively reflect ecological and evolutionary processes that lead to niche expansion or nonexpansion. Niche expansion refers to the proportion of the exotic niche which does not overlap with the native niche. In contrast, niche nonexpansion refers to the proportion of the native niche which does not overlap with the exotic niche. This occurs when species fail to colonize climates in the invaded range that are occupied in the native range [23], often reflecting the fact that species have not had sufficient time to colonize their potential range.

In this study, we use data on the introduced and native populations of the *S. frugiperda* to explore the species’ niche differentiation and potential distribution. The aims of this study are (i) to compare climate spaces occupied by native American and introduced African populations, (ii) to determine whether a climate niche diverged during the invasion of *S. frugiperda* in Africa, and (iii) to predict invasion risk based on four geographic population occurrence records, i.e., native, source, global and African.

## 2. Materials and Methods

### 2.1. Occurrence Data

**Figure 1:**
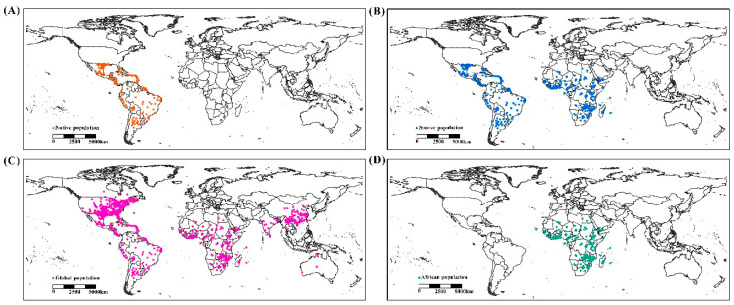
Geographic distribution records of *Spodoptera frugiperda*. (**A**) native population, (**B**) source population, (**C**) global population and (**D**) African population.

Occurrence records were assembled from online databases (Global Biodiversity Information Facility, www.gbif.com), CPC databases (Crop Protection Compendium, https://www.cabi.org/cpc/) and the published literature [9,17,25,26]. These records cover administrative maps, mainly in North America, South America, Africa, Asia and Australia (Figure 1). Records lacking geographic coordinates were georeferenced in Google Maps. A total of 1231 records were attained and filtered at a 2.5 arc grid. Finally, 1146 valid records were used to build the predictive model (Figure 1), comprising 765 native population points, 577 source population points, 1146 global population points and 216 African population points. These were uniformly converted into decimal geographical coordinates for the analysis.

### 2.2. Environmental Variables

Environmental variables were selected by considering their ecological relevance and spatial correlation. Based on 19 bioclimatic variables from the Woldclim (Table S1 [27]), four variables that combined temperature and precipitation were excluded because they displayed artificial discontinuities between adjacent grid cells in some areas [21]. Then, we selected six variables (Pearson < 0.9), i.e., mean diurnal range (Bio2), isothermality (Bio3), max temperature of warmest month (Bio5), precipitation of wettest month (Bio13), precipitation seasonality (Bio15) and precipitation of driest quarter (Bio17). Environmental datasets at a resolution of 2.5 arc minutes were used for the analysis.

### 2.3. Climate Space Comparison

Climate spaces occupied by the native and introduced African populations were compared and mapped in reduced dimensions using PCA (Principal Components Analysis) in Niche A [28]. Niche A can quantify similarity among multiple niches in terms of overlap in n-dimensional environmental spaces [28]. The climate niche suitability during the invasion of *S. frugiperda* was investigated by two alternative hypotheses of equivalency and similarity in statistical tests. We used the “ecospat” package to test whether its climate niche diverged in environmental spaces [29]. This program uses kernel density to fit species’ distribution points and environmental variables associated with each geographical region to test niche suitability. Climate niche overlaps between native and introduced ranges were measured by the first two axes of PCA [30,31]. Schoener’s *D*, which varies between 0 (no overlap at all) and 1 (complete overlap) [32], was used to measure the overall match between the two niches. 

### 2.4. Model Calibration

The four niche models were adopted to map species’ ecological niche and geographic distribution using the popular MaxEnt software (version 3.4.1), the same climatic variables used in the niche shift analyses, and species occurrence records from: (1) native population, (2) global population, (3) source population, and (4) African population. The MaxEnt follows the principle of maximum entropy and spreads out probability as uniformly as possible. The study randomly selected 70% of the native-range points used to fit model; the rest were used for model interpolative and extrapolative evaluations. Partial receiver operating characteristic (ROC) and omission error were used to evaluate model interpolation and transferability, respectively. The traditional AUC approach maybe misleading [33,34]; therefore, we adopted a partial ROC approach, which considers the quality of occurrence points and emphasizes omission error. An AUC ratio of 1 implies that the niche model is no better than a random prediction; the larger the AUC ratio, the better the discrimination ability in the partial ROC approach [33]. 

## 3. Results

### 3.1. Climate Spaces

**Figure 2:**
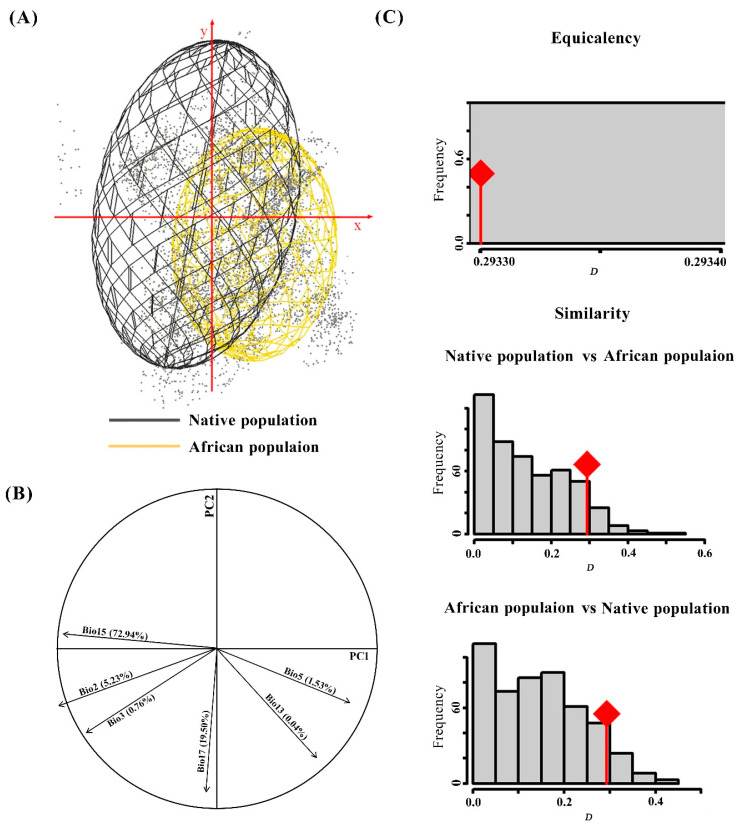
Comparison of *Spodoptera frugiperda* populations between native and Africa. (**A**) Occurrences in a two-dimensional environmental space; axes are the first two principal components; and gray points are the environmental background of the study area. (**B**) The contribution of the climatic variables on the two axes of the PCA and the percentage of each variable. (**C**) Histogram of the niche equivalency and niche similarity tests between native and Africa ranges. The *D* stands for Schoener’s *D* overlap value, red lines with a diamond represent the observed niche overlap, and gray bars represent simulated niche overlaps.

The results showed shifts in the niches of the native and African *S. frugiperda* populations by comparing the ecological space (Figure 2). The contrasting volume size, together with overlaps of climate spaces occupied by the two populations suggested that there were more unoccupied climate spaces in Africa compared with those of the native population (Figure 2A). PCA of climate variables associated with occurrences revealed reduced dimensions that accounted for the observed distribution. The first two components (PC1 and PC2) explained 92.44% of the variance; PC1 was associated with precipitation seasonality (Bio15) and accounted for 72.94%, while PC2 was associated with precipitation of the driest quarter (Bio17), and accounted for 19.50% (Figure 2B).

As seen in panel C, equivalency niche spaces occupied by the native and African populations were Schoener’s *D* = 0.293, meaning that the niche overlap between the two ranges was lower than 30%. This proved that the invaded niche of *S. frugiperda* exhibits both shift and expansion relative to its native range. Actual niche overlaps (red line) were not significantly different from those of the simulated niche overlaps (Figure 2C), indicating that the two populations’ environmental niches were equivalent. This means that there was no niche differentiation during the invasion process of *S. frugiperda*. The equivalency niche results were supported in similarity tests, as actual niche overlap fell into the random simulated overlaps (Figure 2C), indicating that the two populations were not more divergent than expected based on available habitat. A climate niche conservative evaluation further showed that these two populations have not undergone significant alteration in terms of their environmental niche during the invasion process.

### 3.2. Model Predictions

**Figure 3:**
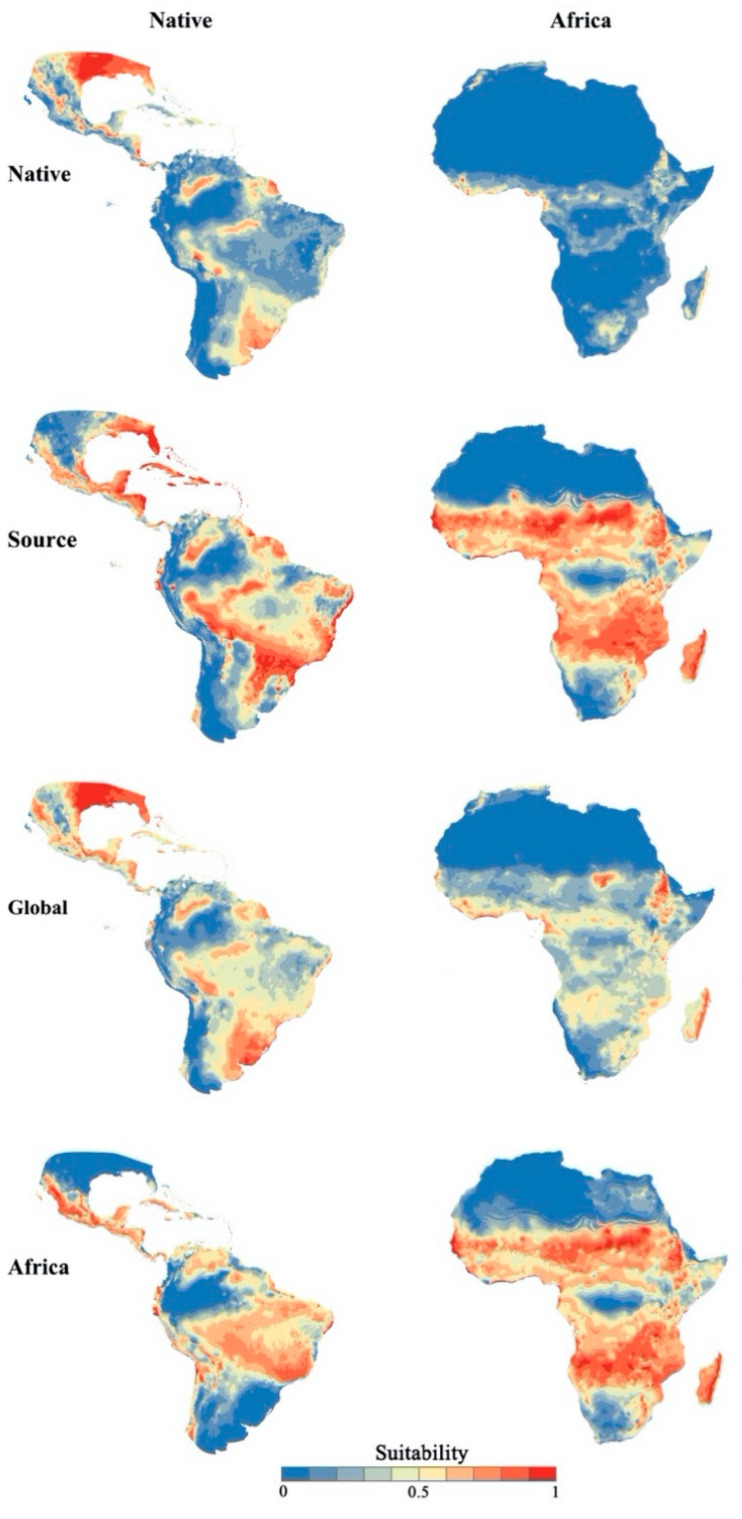
Projections of ecological niche models based on data from the world, source, global and Africa ranges of *Spodoptera frugiperda.*

The results showed that four models could effectively predict the potential distribution of *S. frugiperda* in Africa (Figure 3; Table 1), but there were some differences in the transferability among these models in different areas (Table 1). The areas of potential distribution were properly identified by these models of world distribution, mainly in Americas, Europe, Central Africa, Southern Asia and Central Australia (Figure 3 and Figure S1). These predictions for *S. frugiperda* closely matched its present-day distribution. Among the aforementioned models, the global model presented better performance than the others (Figure 3; Table 1); areas of high suitability were mainly distributed in southwest North America, Central America, South America and Africa. The native model indicated that there is potential for further range expansion in Africa. It showed a more conservative outcome than the other models, and had lower transferability in Africa (Table 1). Moreover, the results showed that *S. frugiperda* was mainly distributed in the southwest of North America, Central America, southern parts of South America, and Africa (Figure 3). In the source and Africa models, predictions for Africa were more liberal than those of the other two models. All predictions showed that *S. frugiperda* was mainly concentrated in countries south of the Sahara Desert (Figure 3).

When niche models were transferred, their performance varied in different areas (Table 1; Figure S2). The four niche model performances were ranked and selected by different modelling approaches for *S. frugiperda*. All niche models showed good performance in the calibration areas (AUC ratio > 1; Table 1). Among these, the global model yielded the best performance, followed by the source and native models. The global model correctly predicted both native and invasive populations (Figure 3). Also, the native model had particularly low spatial transferability in Africa, whereas the source model and Africa model showed good performance in capturing the introduced African occurrence points. Nonetheless, ENMs fitted to occurrence data from the native range of *S. frugiperda* indicated that there was a potential for further range expansion in Africa (Figure 2 and Figure 3).

## 4. Discussion

*S. frugiperda* is native to the tropical and subtropical regions of the Americas. The pest is continuously expanding to new areas and countries. Due to its invasion, economical losses are occurring in food crops, especially in maize production systems in Asia and Africa. The pest is a strong flyer and has a strong ability to migrate long distances in a short time. Between spring and autumn, three successive generations of fall armyworm travelled 1700 km north from Texas and Florida to infest crops as far north as Québec and Ontario [10], causing crop damage and huge economic losses. *S. frugiperda* has rapidly spread throughout most maize-producing areas of Africa since it was introduced in 2016, causing potential economic losses of US $2.481 billion to $6.187 billion per year [33]. As the pest spreads more rapidly and further, crops in northern African countries could be vulnerable to infestations. That is to say, if the *S. frugiperda* could live in the North Africa all year round, then the seasonal migration phenomenon would occur in Europe, which will pose a severe threat to European commercial agriculture. The widespread distribution of *S. frugiperda* in the Americas and Africa indicate that the species is able to migrate north and east while establishing populations and spreading rapidly in Asia. Given the increased trade and transportation among ports in the Americas, Africa and the rest of the world, this could be a huge driving force for further invasions of the *S. frugiperda* into novel areas around the world.

The ecological dimensions and potential distributions of *S. frugiperda* were successfully modeled in this study. Principal components analysis (PCA) was used to reduce the dimension of spatial environment variables, and analyze the limiting effect of each environment variable on geographical distribution [26]. Climate niche evaluation showed that the native population occupied the same ecological niche as the introduced African population (Figure 2C). The contrasting volume size, together with the overlaps, suggested that the species has not colonized the full extent of its native realized niche in its invaded ranges. Therefore, there are suitable climate spaces available that remain unoccupied by the pest (Figure 2A). Little overlap was found between native and introduced ranges, meaning that niche overlap might be influenced by other selective pressures like predators, parasites or trophic chain interactions. Nevertheless, temperature and precipitation are still the main drivers of spatial patterns in large-scale landscapes. All of these results indicate that *S. frugiperda* might have large invasion potential in Africa. Suitable temperature is beneficial to spawning and reproduction, and may create better conditions for its development and further invasion.

It is widely considered that different biogeographic notions are needed to construct a model, which may have a profound impact on that model’s transferability (Figure 3; Table 1). Earlier studies showed that the areas of potential distribution of *S. frugiperda* were mainly in the Americas, Europe, Central Africa, Southern Asia and Central Australia (Figure S1). The native model was built on the basis of population equilibrium and subsequent transfer into introduced areas to predict areas of potential invasion. It has certain advantages in the prediction of the potential distribution of invasive species. It can comprehensively analyze the ecological demand and potential distribution of a given species [35,36]. However, the transferability of the niche model might be limited (Figure 3 and Figure S1). The global model is built on the population nonequilibrium state, and its prediction results are mostly concentrated around the distribution points of the species. It was able to overcome the problem of transferability, and the predicted results were mostly concentrated around the distribution points of the species, even in the absence of independent sample testing [37]. However, if the ecological niche shifts or expands during the invasion, the pooled niche model would be exceedingly useful to predict the distributional potential [38]. Similarly, the source population model not only effectively predicts suitable areas once alien species have established their populations, but can also match the source population with the introduced points to overcome the situation of basic niche differences. Building ENM with data from a species’ invaded range may incorporate phenotypic changes and approximate the fundamental niche; however, this approach is limited by the fact that it assumes that species are in environmental equilibrium [39]. The invaded model could effectively predict the distribution of the species under the condition that the invasive population has already reached a population balance. Therefore, it may be concluded that *S. frugiperda* can survive in harsh climates beyond its native niche limits, and have ability to increase its density when ecological conditions are suitable. Indeed, different methods were used to establish models to explore the potential distribution of *S. frugiperda* which will be of use in the study of the potential distribution of other invasive species.

## 5. Conclusions

*Spodoptera frugiperda* is recognized as one of the most economically important crop pests native to the Americas which has recently invaded Asia and Africa. The prediction results showed that the tropical and subtropical areas of Asia and Africa are most vulnerable, especially the major cultivation areas of maize, rice, sugarcane and other crops. Using multiple introductions to different biogeographic realms in the niche modeling, we were able to successfully plot the patterns of niche expansion and no expansion in the invaded ranges of *S. frugiperda* in Africa. The difference in prediction results was due to the lack of identification of the fundamental and real niches in the four modeling methods. The pest is difficult to control because of its reproductive capacity and rapid and long-distance movement ability, both of which can increase the risk of outbreaks in novel areas. When it reaches new areas with similar environmental conditions to those of its native range, it can further spread and establish an active population. This study confirms the rapid and substantial expansion of the *S. frugiperda* ranges in Africa, and provides an important reference for risk analyses and comprehensive pest management. 

## Figures and Tables

**Table 1:** Interpolative and extrapolative validations of niche model performance.

Validation	Model	Mean AUC ^1^ at 0.95	Mean AUC at 0.5	Ratio ^2^
Interpolative validation	Native model	0.70	0.50	1.40
	Source model	0.83	0.50	1.66
	Global model	0.85	0.50	1.71
	Africa model	0.64	0.50	1.28
Extrapolative validation	Native model to Africa	0.61	0.50	1.22
	Source model to Africa	0.57	0.50	1.14
	Africa model to native ^3^	0.73	0.50	1.55

^1^ Area under the curve; ^2^ Area under the curve ratio; ^3^ Native population range.

## Supplementary Materials

The following are available online at https://www.mdpi.com/2075-4450/11/6/383/s1, Figure S1: World projections of ecological niche models based on data from the native, source, global and Africa ranges of *Spodoptera frugiperda*, Figure S2: Omission rates of native, source and Africa ranges niche models, Table S1: List of bioclimatic characteristics.
