# Supplementary material for: Potential Distribution and Niche Differentiation of Spodoptera frugiperda in Africa
Source: Insects. 2020 Jun 21;11(6):383. doi: 10.3390/insects11060383 (PMC7349815; doi:10.3390/insects11060383)
Supplement: Supplementary file 1 [file insects-11-00383-s001.pdf]

Supplementary

Table S1 List of bioclimatic characteristics.

Bioclimatic characteristics	Abbreviation
Annual Mean Temperature	bio1
Mean Diurnal Range (Mean of monthly (max temp - min temp))	bio2
Isothermality (bio2/bio7) (* 100)	bio3
Temperature Seasonality (standard deviation *100)	bio4
Max Temperature of Warmest Month	bio5
Min Temperature of Coldest Month	bio6
Temperature Annual Range (bio5-bio6)	bio7
Mean Temperature of Wettest Quarter	bio8
Mean Temperature of Driest Quarter	bio9
Mean Temperature of Warmest Quarter	bio10
Mean Temperature of Coldest Quarter	bio11
Annual Precipitation	bio12
Precipitation of Wettest Month	bio13
Precipitation of Driest Month	bio14
Precipitation Seasonality (Coefficient of Variation)	bio15
Precipitation of Wettest Quarter	bio16
Precipitation of Driest Quarter	bio17
Precipitation of Warmest Quarter	bio18
Precipitation of Coldest Quarter	bio19

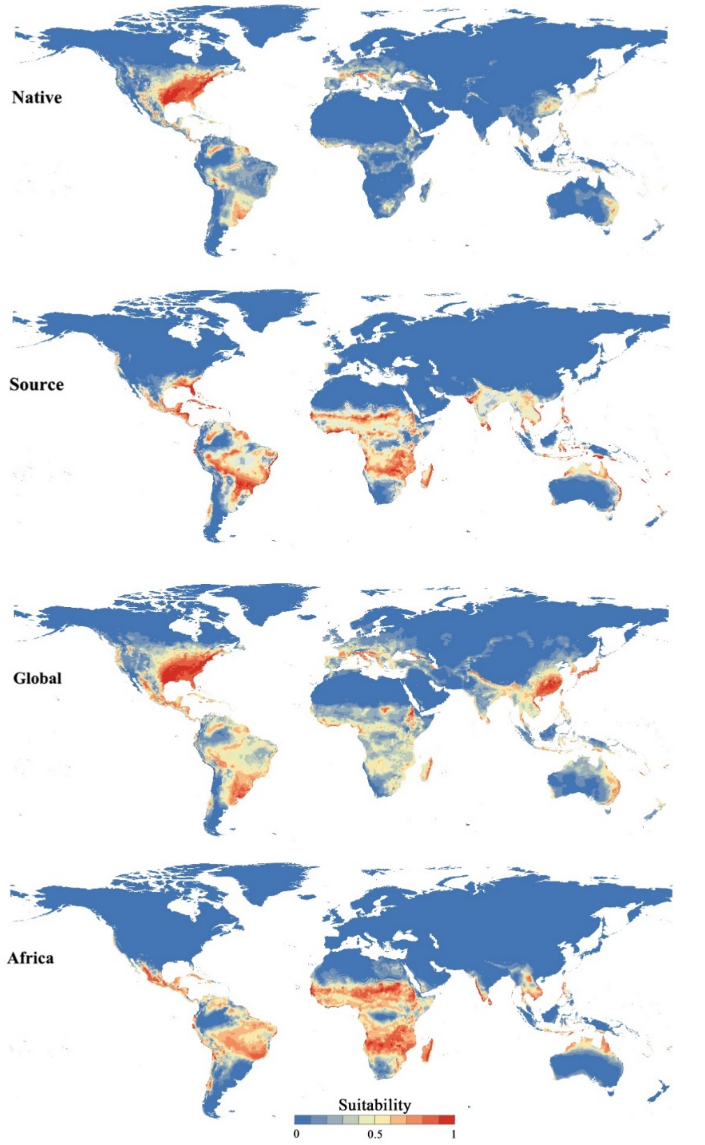

Figure S1. World projections of ecological niche models based on data from the native, source, global and Africa ranges of *Spodoptera frugiperda*.

Deleted: World

Deleted: Source

Deleted: Global

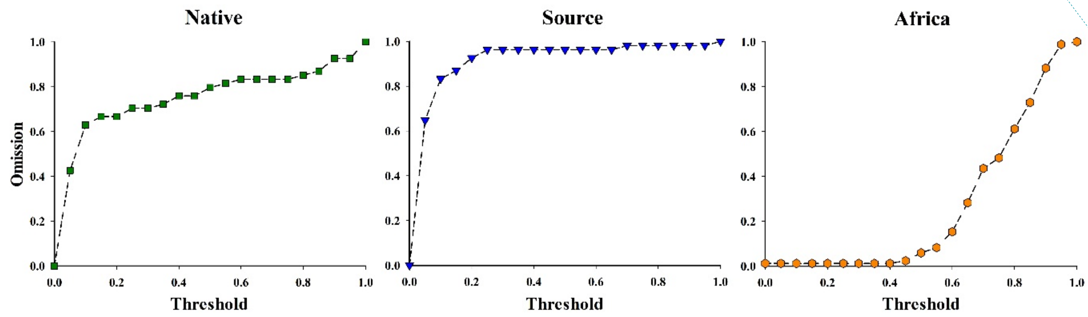

Figure S2. Omission rates of native, source and Africa ranges niche models.

Deleted: Native

Deleted: Source
